# Alternations of Plasma Fatty Acids in Patients With Bipolar Depression Under Acute Treatment of rTMS Combined With Quetiapine and Mood Stabilizer

**DOI:** 10.1002/brb3.70341

**Published:** 2025-02-19

**Authors:** Huan Yu, Xue‐jun Liang, Yu‐ting Qiao, Lin Guo, Zhao‐yang Li, Cui‐hong Zhou, Wen‐jun Wu, Rui Li, Zheng‐wu Peng

**Affiliations:** ^1^ Department of Psychiatry Chang'an Hospital Xi'an China; ^2^ Department of Psychiatry Xijing Hospital Xi'an China; ^3^ Mental Diseases Prevention and Treatment Institute of Chinese PLA No. 988 Hospital of Joint Logistic Support Force Jiaozuo Henan Province China

**Keywords:** acute treatment, bipolar depression, fatty acids, quetiapine combined with mood stabilizers, rTMS

## Abstract

**Purpose:**

Repetitive transcranial magnetic stimulation (rTMS) represents a potential clinical tool in treating bipolar disorder (BD). However, the intervention of rTMS combined with pharmacotherapy on the plasma fatty acids (FAs) in patients with bipolar depression has not been reported yet.

**Method:**

In this study, we assessed the clinical symptoms and evaluated plasma FAs from 30 inpatients with bipolar depression at baseline phase (BD group), after 2 weeks of treatment with rTMS combined with quetiapine and mood stabilizer (BD‐2w group), and 32 healthy controls (HCs).

**Finding:**

We found that acetic acid, propionic acid, isovaleric acid, isobutyric acid, and valeric acid, as well as levels of total short‐chain fatty acids (SCFAs), were decreased in both BD and BD‐2w groups, and levels of several medium‐ and long‐chain fatty acids (MLCFAs) were altered in BD when compared with HC. Moreover, 2 weeks of treatment increased the levels of various MLCFAs. Finally, we developed combinational FAs panels that could distinguish BD from HC (area under the curve [AUC] = 0.961), BD‐2w from HC (AUC = 0.978), and BD from BD‐2w (AUC = 0.891) effectively.

**Conclusion:**

These findings might provide a basis for developing diagnostic methods and reveal the potential relationship between bipolar depression and plasma FAs.

## Graphical abstract

Plasma fatty acids (FAs) were altered in patients with bipolar depression.

Repetitive transcranial magnetic stimulation (rTMS) combined with quetiapine and mood stabilizer alleviated symptoms and changed plasma FAs composition in patients.

We identified FAs panels, which can distinguish bipolar disorder (BD) from healthy control (HC), BD‐2w from HC, and BD‐2w from BD as well.

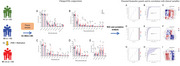

## 1 Introduction

Bipolar disorder (BD) is a severe mental disorder that affects approximately 40 million individuals, ranking as the 17th leading source of disability among all diseases worldwide (Vigo, Thornicroft, and Atun 2016). The global prevalence of BD is approximately 2% (Merikangas et al. 2011) and its lifetime prevalence is 2.5% in men and 2.3% in women (McGrath et al. 2023). BD is associated with a higher risk of suicide, about 34% of patients with BD attempt suicide and 15%–20% die by suicide (Beyer and Weisler 2016; Dong et al. 2019; Plans et al. 2019) and the treatment compliance of BD patients is affected by some factors, such as substance use disorder and illness severity (Pompili et al. 2013). Moreover, BD is always combined with other psychiatric and cardiovascular diseases, such as anxiety disorders, substance use, hypertension, and diabetes (Crump et al. 2013; Hossain et al. 2019; Krishnan 2005). Manic or hypomanic episodes are the defining feature of BD, depression is the initial presentation in most patients which is one of the main reasons that nearly 40% of patients with BD are misdiagnosed as depression (Hirschfeld, Lewis, and Vornik 2003; Vieta and Suppes 2008). In addition, although most patients with BD have clinical features, accurate diagnosis can only be obtained after the first contact with primary healthcare providers for about 6–10 years (Dagani et al. 2017). Therefore, it is necessary to diagnose BD accurately and timely.

Several factors have been reported to be involved in the etiopathogenesis of BD, such as alternations in genetic, physiological, and immunoinflammatory (Dadouli et al. 2022; Scaini et al. 2020). Importantly, fatty acids (FAs) are essential in energy metabolism, immunoregulation, and signaling pathways (Panezai and van Dyke 2023; Zhang et al. 2023). FAs are classified as short‐chain fatty acids (SCFAs, a chain length from one to six carbons), and medium‐ and long‐chain fatty acids (MLCFAs, a chain length of more than eight carbons). SCFAs are mainly produced by the fecal microbiota fermentation of the dietary fiber whereas MLCFAs are primarily derived from dietary triglycerides (TGs). Despite their role as energy sources, increasing evidence indicates that FAs, mainly free FAs, are also natural ligands for a series of G protein‐coupled receptors (GPCRs), which interweave metabolism and immunity in many ways (Kimura et al. 2020). Recently, the role of FAs in psychiatric disorders has been reported. For example, peripheral FAs were altered in patients with schizophrenia and major depressive disorder (MDD; Liu et al. 2023). Furthermore, targeting FAs intake, especially omega‐3 polyunsaturated fatty acids (N3 PUFAs) might have potential benefits for the treatment of schizophrenia and MDD (Borsini et al. 2021; Goh et al. 2021). Correspondingly, the potential role of FAs in BD has also been revealed. Previous work found that plasma docosahexaenoic acid (DHA) was decreased while arachidonic acid (AA), eicosapentaenoic acid (EPA), and alpha‐linolenic acid (ALA) were increased in bipolar patients when compared with healthy controls (HCs; Pomponi et al. 2013). Recent studies also indicated that N3 PUFAs may be protective against BD and developmental N3 PUFA deficiency might be a risk factor for BD, whereas omega‐6 (N6) PUFAs and an increased ratio of N6:N3 may increase the risk of BD (McNamara et al. 2022; Zhang et al. 2023). Moreover, dietary intake or supplementation of PUFAs, especially N3 PUFAs seems to improve symptoms of BD (Gabriel et al. 2023). Nevertheless, the differences of FAs between patients with BD and healthy individuals, as well as the impact of repetitive transcranial magnetic stimulation (rTMS) combined with pharmacotherapy on the composition of plasma FAs, still need further elucidation.

Pharmacotherapy is the mainstay of treatment which should be adjusted according to the clinical manifestations of patients (hypomania/mania or depression; Baldessarini, Tondo, and Vazquez 2019). The main goal is to alleviate the symptoms of the current emotional episode, reduce the severity, and limit the number of future episodes. Combination therapy with atypical antipsychotic drugs, such as quetiapine, associated with a mood stabilizer, such as valproate, lithium, and lamotrigine, is recommended for both acute and long‐term treatment of BD in the majority of clinical practice guidelines (Kishi et al. 2022; Nestsiarovich et al. 2022; Yildiz et al. 2023). Recently, rTMS has been suggested as a treatment option for BD, especially for bipolar depression (Gold et al. 2019). The high‐frequency rTMS applied for the left dorsolateral prefrontal cortex (DLPFC) not only increased the response rates but also improved the processing speed and working memory of BD patients (Nguyen et al. 2021; Yang et al. 2019). Although our previous work found that rTMS could influence plasma FAs in patients with depression (Li et al. 2024), thus far, no study has reported the effect of rTMS combined with pharmacotherapy on plasma FA composition in patients with bipolar depression.

Considering the above, we hypothesized that the changes of FAs in peripheral blood may also participate in the pathogenesis of BD, and the alternations of specific FAs may be one of the molecular mechanisms of rTMS combined with pharmacotherapy. We detected the concentrations of plasma FAs from adult hospitalized patients with bipolar depression at baseline (BD, *n* = 30) and 2 weeks after rTMS combined with quetiapine and mood stabilizer treatment (BD‐2w, *n* = 30) and 32 gender‐ and age‐matched HCs. Meanwhile, we explored the characteristic changes of FAs among BD, BD‐2w, and HC, and developed the potential biomarkers of FAs that can distinguish BD and HC, BD‐2w, and HC, as well as BD and BD‐2w.

## 2 Methods

### 2.1 Participants

The diagnosis of BD was performed by a structured clinical interview based on the Diagnostic and Statistical Manual (DSM‐5) of Mental Disorders criteria. The Bech–Rafaelsen Mania Rating Scale (BRMS), 17‐item Hamilton Depression Rating Scale (HAMD), and Hamilton Anxiety Scale (HAMA) were used to assess the severity of symptoms. The clinical interview and scale evaluation were independently administered by senior psychiatrists who knew nothing about clinical trial design. The detailed pharmacotherapeutic combination and dose are presented in Supporting Information Table 1. The exclusion criteria were pregnancy, lactation, or menstrual period; hypertension; a severely imbalanced diet, such as high‐fat diet preferences; obesity, which was defined as a body mass index (BMI) ≥ 28.0; illicit drug use; diabetes; diseases of the digestive system; alcohol abuse or dependence; taking other antipsychotics; unable to complete 2‐week treatment or discharged early. Blood samples were collected and the plasma was obtained under fasting conditions between 8 am and 10 am. The blood lipids, including low‐density lipoprotein (LDL), high‐density lipoprotein (HDL), cholesterol (CHOL), TG, and blood glucose (GLU) were also detected.

### 2.2 rTMS

As described previously (Yang et al. 2019), treatment was administered using a TMS‐100 magnetic stimulator for 14 successive days (Max, Zhengzhou Medical Technology Company Ltd., China). The resting motor threshold (MT) was determined and patients received 10‐Hz rTMS (3000 pulses daily, delivered at 110% MT at 30‐s intertrain intervals) to the left DLPFC.

### 2.3 SCFAs and MLCFAs Measurement

The SCFAs and MLCFAs standard solution, MLCFAs extraction and sample were prepared as described previously (Li et al. 2024). Plasma SCFAs were detected by liquid chromatography–mass spectrometry (LC/MS). The LC–ESI–MS/MS (UHPLC‐Qtrap) was used as described previously (Phulpoto et al. 2024). The default parameters are used and manual inspection is assisted for identifying and integrating ion fragments. Then draw the linear regression standard curve and calculate the concentration results. Plasma MLCFAs were detected by gas chromatography–mass spectrometry (GC–MS). Gas chromatography (Agilent 8890b) and mass selective detector (Agilent 5977b/7000d) were used for analysis (Majorbio Pharm Technology Co. Ltd., Shanghai, China). The Masshunter quantitative software (Agilent, USA, version number: v10.0.707.0) was used to analyze the GC–MS raw data with default parameters to automatically identify and integrate all ion fragments, and manually check all integrations.

### 2.4 Statistical Analyses

Statistical analyses were performed by using R‐3.5.3 and SPSS 21.0. Except for the comparison of gender (chi‐squared analysis), the continuous variables were analyzed by the Shapiro–Wilk test. Then the normal distributed data were analyzed by using independent or paired samples *t*‐test (represented by mean ± standard deviation [SD]), and the abnormally distributed data, which was represented by M (P25, P75), was analyzed by using the Wilcoxon test or Mann–Whitney *U* test. MetaboAnalyst 5.0 (online software, https://www.metaboanalyst.ca/MetaboAnalyst/) was used to conduct random forest analysis and draw receiver operating characteristic (ROC) curves. The top 10 FAs with the greatest differences were selected as the candidate biomarker panel and correlations between the levels of these FAs and clinical parameters were assessed by Spearman correlation analysis.

## 3 Results

### 3.1 Clinical Data Evaluation

There were no significant differences between BD and HC in gender (*p* = 0.213), age (*p* = 0.767), BMI (*p* = 0.130), GLU (*p* = 0.260) and HDL (*p* = 0.163). However, the scores of HAMA (*p* = 0.014) and HAMD (*p* < 0.01), and the level of LDL (*p* = 0.001) were increased while the level of TG (*p* = 0.005) and CHOL (*p* = 0.024) were decreased in the BD group in comparison with the HC group (Table 1). There were no significant differences between BD‐2w and HC groups in terms of BMI (*p* = 0.105), TG (*p* = 0.172), the scores of HAMA (*p* = 0.091) and CHOL (*p* = 0.166). However, levels of HDL (*p* = 0.049) and GLU (*p* = 0.021) were decreased whereas scores of HAMD (*p* = 0.027) and levels of LDL were increased in the BD‐2w when compared with the HC group (Table 2). Furthermore, we also compared the clinical data between the BD and BD‐2w groups. There were no significant differences between BD and BD‐2w in terms of TG (*p* = 0.199), LDL (*p* = 0.067) and CHOL (*p* = 0.102). However, the scores of HAMA, BRMS, and HAMD (all *p* < 0.01), and levels of HDL and GLU (all *p* < 0.05) were decreased in the BD‐2w group in comparison with the BD group (Table 3). Detailed clinical and demographic characteristics are shown in Supporting Information Table 1.

**TABLE 1:** Comparison of clinical characteristics data and symptom scale assessment between the healthy control (HC) and bipolar disorder (BD) groups.

Variable	HC (*n* = 32)	BD (*n* = 30)	*χ*2/*Z*/*t*	p value
Gender (male/female)a	10/22	14/16	*χ*2 = 1.551	0.213
Age [years, M (P25, P75)]b	29.00 (25.00, 36.75)	32.00 (26.00, 45.00)	*Z* = −2.967	0.767
BMI [M (P25, P75)]b	20.70 (18.99, 23.48)	21.46 (19.87, 23.55)	*Z* = −1.514	0.130
GLU [M (P25, P75)]b	4.94 (4.42, 5.52)	4.69 (4.45, 4.96)	*Z* = −1.127	0.260
TG [M (P25, P75)]b	1.46 (1.11, 1.82)	0.87 (0.75, 1.39)	*Z* = −2.825	0.005
LDL [M (P25, P75)]b	1.66 (1.15, 2.10)	2.20 (1.88, 2.69)	*Z* = −3.353	0.001
HAMA [M (P25, P75)]b	4.50 (2.75, 6.25)	18.50 (4.00, 22.75)	*Z* = −2.447	0.014
HAMD [M (P25, P75)]b	3.00 (2.00, 5.00)	19.00 (3.25, 27.00)	*Z* = −3.561	< 0.001
HDLc	1.29 ± 0.08	1.21 ± 0.28	*t* = 1.427	0.163
CHOLc	4.22 ± 1.16	3.68 ± 0.58	*t* = 2.336	0.024

*Note*: ^a^Chi‐square tests; ^b^Mann–Whitney *U* test; ^c^Independent sample *t*‐test; Values are shown as mean ± SD or *M* (*P*_25_, *P*_75_).

Abbreviations: BMI, body mass index; CHOL, cholesterol; GLU, blood glucose; HAMA, Hamilton Anxiety Scale; HAMD, Hamilton Depression Rating Scale; HDL, high‐density lipoprotein; LDL, low‐density lipoprotein; SD, standard deviation; TG, triglyceride.

**TABLE 2:** Comparison of clinical characteristics data and symptom scale assessment between the healthy control (HC) and BD‐2w groups.

Variable	HC (*n* = 32)	BD‐2w (*n* = 30)	*Z*/*t*	p value
BMI [M (P25, P75)]a	20.70 (18.99, 23.48)	21.46 (20.03, 23.69)	*Z* = −1.620	0.105
TG [M (P25, P75)]a	1.46 (1.11, 1.82)	1.32 (0.75, 1.69)	*Z* = −1.367	0.172
HDL [M (P25, P75)]a	1.28 (1.22, 1.37)	1.17 (1.08, 1.47)	*Z* = −1.966	0.049
HAMA [M (P25, P75)]a	4.50 (2.75, 6.25)	9.5 (2.00, 15.00)	*Z* = −1.691	0.091
HAMD [M (P25, P75)]a	3.00 (2.00, 5.00)	12.00 (2.00, 14.75)	*Z* = −2.216	0.027
GLUb	4.96 ± 0.64	4.63 ± 0.42	*t* = 2.371	0.021
LDLb	1.66 ± 0.67	2.32 ± 0.54	*t* = −4.220	< 0.001
CHOLb	4.22 ± 1.16	3.88 ± 0.67	*t* = 1.406	0.166

*Note*: ^a^Mann–Whitney *U* test; ^b^Independent sample *t*‐test; Values are shown as mean ± SD or *M* (*P*_25_, *P*_75_).

Abbreviations: BMI, body mass index; CHOL, cholesterol; GLU, blood glucose; HAMA, Hamilton Anxiety Scale; HAMD, Hamilton Depression Rating Scale; HDL, high‐density lipoprotein; LDL, low‐density lipoprotein; SD, standard deviation; TG, triglyceride.

**TABLE 3:** Comparison of clinical characteristics data and symptom scale assessment between the bipolar disorder (BD) and BD‐2w groups.

Variable	BD (*n* = 30)	BD‐2w (*n* = 30)	*Z*/*t*	p value
TG [M (P25, P75)]a	0.87 (0.75, 1.39)	1.32 (0.75, 1.69)	*Z* = −1.286	0.199
HDL [M (P25, P75)]a	1.19 (0.98, 1.47)	1.17 (1.08, 1.47)	*Z* = −2.006	0.045
HAMA [M (P25, P75)]a	18.50 (4.00, 22.75)	9.5 (2.00, 15.00)	*Z* = −4.291	< 0.001
HAMD [M (P25, P75)]a	19.00 (3.25, 27.00)	12.00 (2.00, 14.75)	*Z* = −4.461	< 0.001
GLU [M (P25, P75)]a	4.69 (4.45, 4.96)	4.52 (4.34, 4.91)	*Z* = −2.228	0.026
LDL [M (P25, P75)]a	2.20 (1.88, 2.69)	2.28 (1.95, 2.65)	*Z* = −0.514	0.607
BRMS	14.00 (5.00, 17.75)	6.50 (2.25, 13.00)	*Z* = −4.375	< 0.001
CHOLb	3.68 ± 0.58	3.88 ± 0.67	*t* = −1.686	0.102

*Note*: ^a^Wilcoxon test; ^b^Paired sample *t*‐test; Values are shown as mean ± SD or *M* (*P*_25_, *P*_75_).

Abbreviations: BRMS, Bech–Rafaelsen Mania Rating Scale; CHOL, cholesterol; GLU, blood glucose; HAMA, Hamilton Anxiety Scale; HAMD, Hamilton Depression Rating Scale; HDL, high‐density lipoprotein; LDL, low‐density lipoprotein; SD, standard deviation; TG, triglyceride.

### 3.2 Comparison of SCFAs in BD, BD‐2w, and HC

**FIGURE 1:**
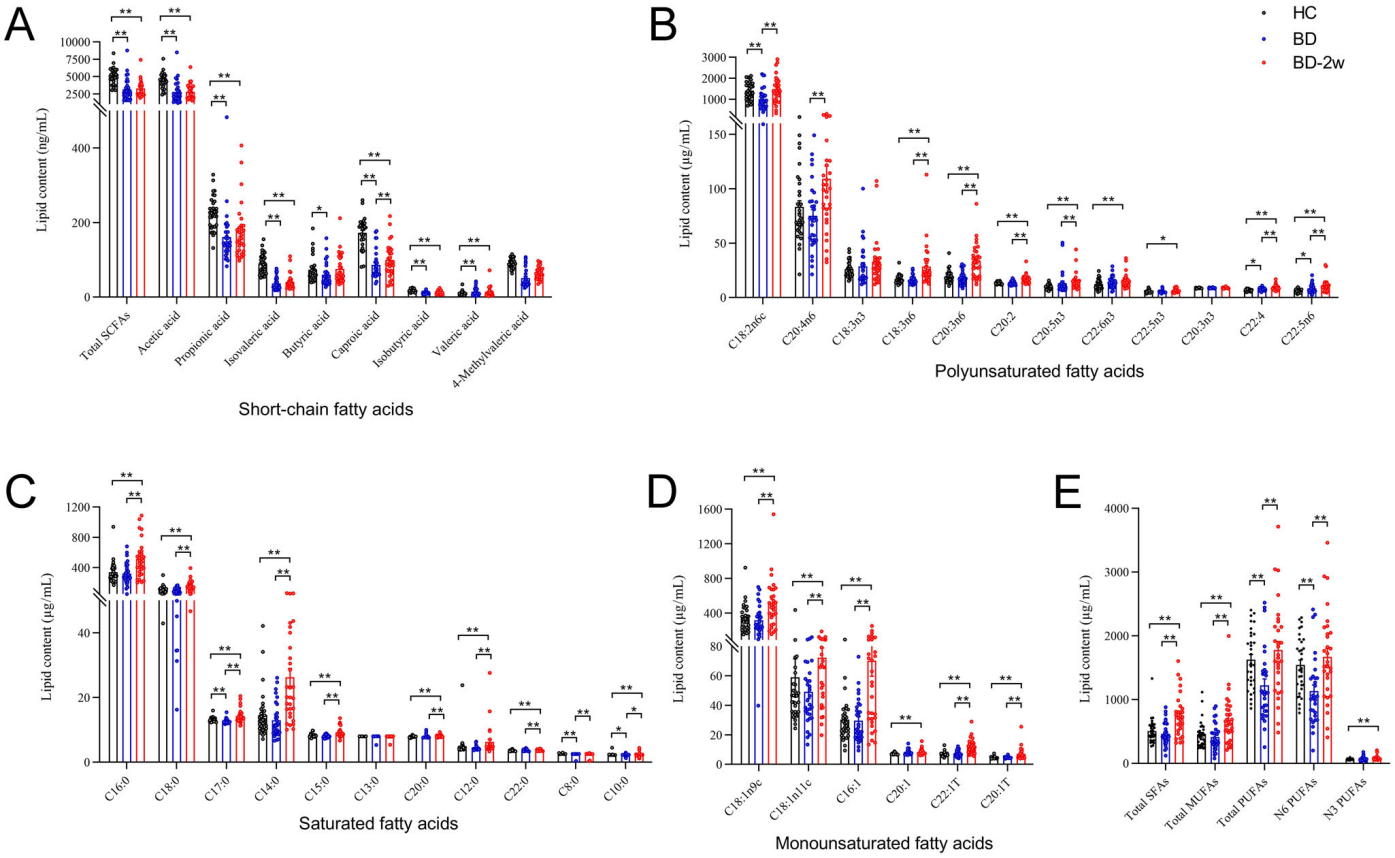
The differential concentration of fatty acids (FAs) among healthy control (HC), bipolar disorder (BD), and BD‐2w participants. (A) Short‐chain fatty acids, (B) polyunsaturated fatty acids, (C) saturated fatty acids, (D) monounsaturated fatty acids, (E) total SFAs, total MUFAs, total PUFAs, total N6‐PUFAs, and total N3‐PUFAs. The black circle, blue circle, and red circle represent one value from an individual in the HC, BD, and BD‐2w, respectively. C18:2n6c, cis‐linoleic acid; C20:4n6, arachidonic acid (AA); C18:3n3, α‐linolenic acid; C18:3n6, gamma‐linolenic acid; C20:3n6, gamma dihomo linoleic acid; C20:2, 11,14‐eicosadienoate; C20:5n3, eicosapentaenoic acid (EPA); C22:6n3, docosahexaenoic acid (DHA); C22:5n3, docosapentaenoic acid (DPA); C20:3n3, eicosatrienoic acid; C22:4, docosatetraenoate; C22:5n6, docosapentaenoic acid; C16:0, palmitic acid; C18:0, stearic acid; C17:0, heptadecanoic acid; C14:0, myristic acid; C15:0, pentadecanoic acid; C13:0, tridecanoic acid; C20:0, arachidic acid; C12:0, lauric acid; C22:0, behenic acid; C8:0, caprylic acid; C10:0, decanoate acid; 18:1n9c, cis‐oleic acid; C18:1n11c, cis‐vaccenic acid; C16:1, palmitoleic acid; C20:1, eicosenoic acid; C22:1T, trans‐erucic acid; C20:1T, trans‐eicosenoic acid; MUFAs, monounsaturated fatty acids; N3 PUFAs, omega‐3 polyunsaturated fatty acids; N6 PUFAs, omega‐6 polyunsaturated fatty acids; PUFAs, polyunsaturated fatty acids; SFAs, saturated fatty acids. *p < 0.05; **p < 0.01.

Except for 4‐methylvaleric acid, concentrations of total SCFAs (*Z* = −5.141, *p* < 0.001), acetic acid (*Z* = −4.9916, *p* < 0.001), propionic acid (*Z* = −4.043, *p* < 0.001), isovaleric acid (*Z* = −5.057, *p* < 0.001), butyric acid (*Z* = −2.485, *p* < 0.05), caproic acid (*Z* = −5.254, *p* < 0.001), isobutyric acid (*Z* = −6.142, *p* < 0.001), and valeric acid (*Z* = −5.410, *p* < 0.001) were decreased in BD than that in the HC group. Similarly, concentrations of total SCFAs (*Z* = −4.916, *p* < 0.001), acetic acid (*Z* = −4.761, *p* < 0.001), propionic acid (*Z* = −4.972, *p* < 0.001), isovaleric acid (*Z* = −5.798, *p* < 0.001), caproic acid (*Z* = −5.353, *p* < 0.001), isobutyric acid (*Z* = −5.888, *p* < 0.001), and valeric acid (*Z* = −4.219, *p* < 0.001) were decreased in BD‐2w than that in the HC group. Wilcoxon test or paired samples *t*‐test showed that there were no significant differences between BD and BD‐2w in terms of total SCFAs (*Z* = −1.059, *p* = 0.289), acetic acid (*Z* = −0.998, *p* = 0.318), propionic acid (*Z* = −1.306, *p* = 0.192), isovaleric acid (*Z* = −1.224, *p* = 0.221), isobutyric acid (*Z* = −1.296, *p* = 0.159), butyric acid (*Z* = −1.872, *p* = 0.061), 4‐methylvaleric acid (*Z* = −0.936, *p* = 0.349), and valeric acid (*t* = −1.152, *p* = 0.165). Only levels of caproic acid (*Z* = −3.189, *p* < 0.001) were increased in BD‐2w than that in the BD group. It suggests that most plasma SCFAs were decreased in BD patients. However, only the levels of capronic acid and butyric acid were increased after 2 weeks of pharmacotherapy (Figure 1A).

### 3.3 Comparison of PUFAs in BD, BD‐2w, and HC

There were no significant differences between BD and HC groups in the concentrations of C18:3n6 (*Z* = −1.690, *p* = 0.091), C18:3n3 (*Z* = −1.211, *p* = 0.226), C20:2 (*Z* = −0.958, *p* = 0.338), C20:3n3 (*Z* = −0.846, *p* = 0.398), C20:4n6 (*Z* = −0.887, *p* = 0.375), C20:5n3 (*Z* = −1.155, *p* = 0.248), C22:5n3 (*Z* = −0.324, *p* = 0.746), C20:3n6 (*Z* = −0.662, *p* = 0.508), and C22:6n3 (*t* = −1.362, *p* = 0.178). In addition, levels of C22:4 (*t* = −2.185, *p* = 0.034) and C22:5n6 (*t* = −3.221, *p* = 0.003) were increased whereas levels of C18:2n6c (*t* = 3.413, *p* = 0.001) were decreased in BD than that in the HC group. Moreover, there were no significant differences between BD‐2w and HC groups in the concentrations of C18:3n3 (*Z* = −1.211, *p* = 0.226), C20:3n3 (*Z* = −0.127, *p* = 0.899), C20:4n6 (*Z* = −1.902, *p* = 0.057), C18:2n6c (*t* = −0.537, *p* = 0.598). Whereas levels of C18:3n6 (*Z* = −4.155, *p* < 0.001), C20:2 (*Z* = −4.324, *p* < 0.001), C20:5n3 (*Z* = −4.240, *p* < 0.001), C22:5n3 (*Z* = −2.381, *p* = 0.017), C22:5n6 (*Z* = −5.324, *p* < 0.001), C22:6n3 (*Z* = −2.634, *p* = 0.008), C22:4 (*t* = −5.458, *p* < 0.001), and C20:3n6 (*t* = −4.871, *p* < 0.001) were increased in BD‐2w than that in the HC group. Furthermore, paired sample tests found that levels of C18:3n6 (*Z* = −3.898, *p* < 0.001), C20:2 (*Z* = −4.247, *p* < 0.001), C20:4n6 (*Z* = −2.705, *p* = 0.007), C20:5n3 (*Z* = −2.869, *p* = 0.004), C22:5n3 (*Z* = −3.075, *p* = 0.002), C22:5n6 (*Z* = −3.034, *p* = 0.002), C20:3n6 (*Z* = −4.268, *p* < 0.001), C18:2n6c (*t* = −3.442, *p* = 0.002), and C22:4 (*t* = −4.220, *p* < 0.001) were increased in BD‐2w than that in the BD group (Figure 1B).

In the comparison of the HC group, concentrations of total PUFAs (*Z* = −3.113, *p* = 0.002) and total N6 PUFAs (*t* = 3.278, *p* = 0.002) were decreased in the BD group, and the total N3 PUFAs (*Z* = −2.888, *p* = 0.004) were increased in BD‐2w group. Moreover, concentrations of total PUFAs (*Z* = −2.394, *p* = 0.003) and total N6 PUFAs (*t* = −3.540, *p* = 0.001) were decreased in the BD group when compared with the BD‐2w group (Figure 1E). It suggests that plasma PUFAs and N6 PUFAs were decreased in patients with BD, quetiapine combined with mood stabilizers can not only improve this phenomenon but also increase the level of N3 PUFAs.

### 3.4 Comparison of SFAs and MUFAs in BD, BD‐2w, and HC

There were no significant differences between BD and HC groups in the concentrations of C12:0 (*Z* = −1.648, *p* = 0.099), C13:0 (*Z* = −1.121, *p* = 0.262), C14:0 (*Z* = −1.254, *p* = 0.210), C20:0 (*Z* = −1.254, *p* = 0.210), C22:0 (*Z* = −1.317, *p* = 0.188), C16:0 (*t* = 0.512, *p* = 0.610), and C15:0 (*t* = 1.884, *p* = 0.064). However, levels of C10:0 (*Z* = −2.015, *p* = 0.044), C17:0 (*Z* = −2.599, *p* = 0.009), and C8:0 (*Z* = −2.854, *p* = 0.004) were decreased in BD than that in the HC group. Moreover, levels of C10:0 (*Z* = −2.642, *p* = 0.008), C12:0 (*Z* = −2.704, *p* = 0.007), C14:0 (*Z* = −3.888, *p* < 0.001), C17:0 (*Z* = −2.704, *p* = 0.007), C20:0 (*Z* = −2.789, *p* = 0.005), C22:0 (*Z* = −3.613, *p* < 0.001), C15:0 (*Z* = −3.324, *p* < 0.001), C16:0 (*t* = −3.529, *p* < 0.001), C18:0 (*t* = −3.004, *p* = 0.005) were increased in BD‐2w than that in the HC group. Paired sample tests further found that levels of C8:0 (*Z* = −2.952, *p* = 0.003), C10:0 (*Z* = −2.499, *p* = 0.012), C12:0 (*Z* = −2.982, *p* = 0.003), C14:0 (*Z* = −4.350, *p* < 0.001), C15:0 (*Z* = −3.630, *p* < 0.001), C17:0 (*Z* = −3.507, *p* < 0.001), C20:0 (*Z* = −2.581, *p* = 0.010), C22:0 (*Z* = −2.551, *p* = 0.011), C16:0 (*t* = −4.480, *p* < 0.001), C18:0 (*t* = −4.444, *p* < 0.001) were increased in BD‐2w than that in the BD group (Figure 1C).

No significant differences between BD and HC groups were observed in terms of C16:1 (*Z* = −0.310, *p* = 0.757), C18:1n11c (*Z* = −0.225, *p* = 0.822), C20:1T (*Z* = −0.662, *p* = 0.508), C20:1 (*Z* = −0.620, *p* = 0.535), C22:1T (*Z* = −0.349, *p* = 0.693), and C18:1n9c (*t* = 0.530, *p* = 0.598). However, levels of C16:1 (*Z* = −3.296, *p* < 0.001), C18:1n11c (*Z* = −2.817, *p* = 0.005), C20:1T (*Z* = −4.578, *p* < 0.001), C20:1 (*Z* = −3.867, *p* < 0.001), C22:1T (*Z* = −4.817, *p* < 0.001), and C18:1n9c (*t* = −3.016, *p* = 0.004) were increased in BD‐2w than that in the HC group. These MUFAs, except C20:1, were also increased in BD‐2w than that in the BD group (Figure 1D). Furthermore, in comparison of the HC group, concentrations of total SFAs (*t* = −3.055, *p* = 0.004) and total MUFAs (*Z* = −3.455, *p* = 0.001) were increased in the BD‐2w group. Similarly, both total SFAs (*t* = −4.627, *p* < 0.001) and total MUFAs (*Z* = −3.260, *p* = 0.001) were increased in the BD‐2w group when compared with the BD group (Figure 1E). Together, quetiapine combined with mood stabilizers intervention upregulates a large amount of SFAs and MUFAs levels in patients, while there were fewer changes between the BD and HC groups.

### 3.5 Characteristic Plasma FAs Between BD and HC, BD‐2w and HC, and BD and BD‐2w

**FIGURE 2:**
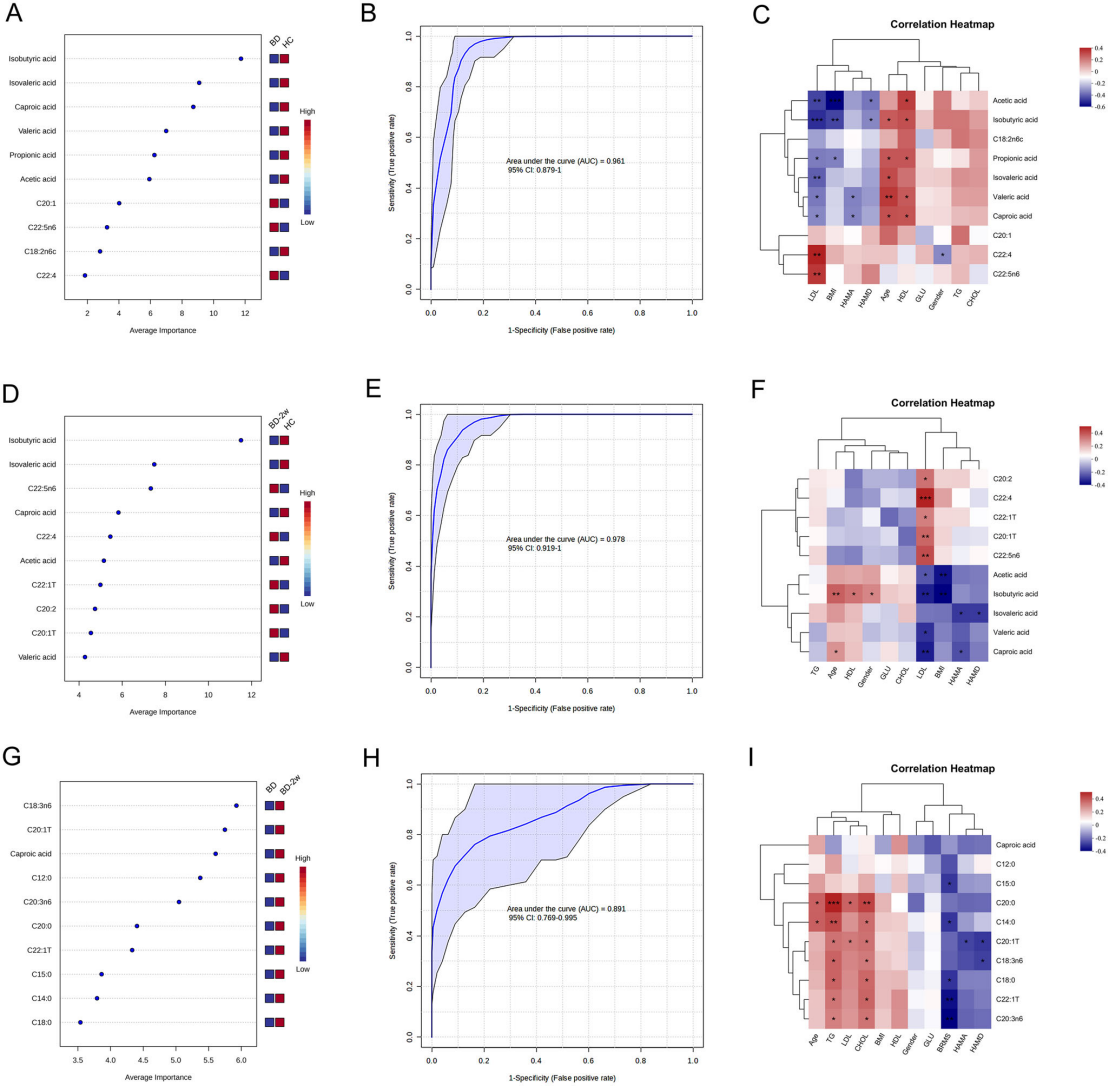
Potential biomarker panels and the correlation between levels of identified fatty acid (FA) species and clinical variables. Top 10 differential FAs identified from random forest classifiers, and area under the curve (AUC) value of receiver operating characteristic (ROC) analysis for discriminating the bipolar disorder (BD) from healthy control (HC) (A, B), BD‐2w from HC (D, E), and BD from BD‐2w (G, H). (C, F, and I) Correlation between concentrations of identified FA species and levels of LDL, BMI, HAMA, HAMD or BRMS, Age, HDL, GLU, gender, TG, and CHOL. Red and blue squares indicate positive and negative correlations, respectively, and the intensities of the colors are proportional to the degree of correlation. BMI, body mass index; BRMS, Bech–Rafaelsen Mania Rating Scale; CHOL, cholesterol; CI, confidence interval; GLU, blood glucose; HAMA, Hamilton Anxiety Scale; HAMD, Hamilton Depression Rating Scale; HDL, high‐density lipoprotein; LDL, low‐density lipoprotein; TG, triglyceride. *p < 0.05; **p < 0.01; ***p < 0.001.

Differential FAs between BD and HC groups including isobutyric acid, isovaleric acid, caproic acid, valeric acid, acetic acid, propionic acid, C20:1, C22:5n6, C18:2n6c, and C22:4 were selected as a panel that can distinguish BD from HC effectively (area under the curve [AUC] = 0.961, Figure 2A,B). Moreover, concentrations of acetic acid, isovaleric acid, propionic acid, caproic acid, isobutyric acid, and valeric acid were negatively correlated while concentrations of C22:5n6 and C22:4 were positively correlated with the content of LDL. Levels of acetic acid and isobutyric acid were also negatively correlated with BMI and scores of HAMD, and only levels of caproic acid and valeric acid were negatively correlated with scores of HAMA (Figure 2C). Moreover, a panel containing isobutyric acid, isovaleric acid, C22:5n6, C22:4, caproic acid, acetic acid, C22:1T, C20:2, C20:1T, and valeric acid could effectively distinguish BD‐2w and HC (AUC = 0.978, Figure 2D,E). Levels of C22:5n6, C22:1T, C22:4, C20:1T, and C20:2 were positively correlated whereas levels of isobutyric acid, caproic acid, valeric acid, and acetic acid were negatively correlated with levels of LDL. Concentrations of acetic acid and isobutyric acid were negatively correlated with BMI, concentrations of isovaleric acid were negatively correlated with scores of HAMD and HAMA, and levels of caproic acid were negatively correlated with scores of HAMA (Figure 2F). Finally, a panel consisting of C18:3n6, C20:1T, caproic acid, C12:0, C20:3n6, C20:0, C22:1T, C15:0, C14:0, and C18:0 distinguish BD and BD‐2w (AUC = 0.891, Figure 2G,H). Levels of C15:0, C14:0, C18:0, C22:1T, and C20:3n6 were negatively correlated with scores of BRMS, while levels of C20:0, C14:0, C18:3n6, C20:1T, C18:0, C22:1T, and C20:3n6 were positively correlated with TG and CHOL. Content of C20:1T was negatively correlated with scores of HAMD and HAMA, and levels of C18:3n6 were negatively correlated with scores of HAMD (Figure 2I). These results indicated that the decreased content of isobutyric acid, isovaleric acid, caproic acid, propionic acid, valeric acid, acetic acid, and C18:2n6c, and increased levels of C20:1, C22:5n6, and C22:4 might be a potential feature that could effectively distinguish BD from HC. Meanwhile, the decreased levels of isobutyric acid, isovaleric acid, caproic acid, acetic acid, and valeric acid, and increased levels of C22:5n6, C22:4, C22:1T, C20:2, and C20:1T might be a potential feature that could effectively distinguish BD‐2w from HC. Whereas the increased levels of C18:3n6, C20:1T, caproic acid, C12:0, C20:3n6, C20:0, C22:1T, C15:0, C14:0, and C18:0 might be a potential biomarker that could effectively distinguish BD‐2w from BD.

## 4 Discussion

In this study, we elucidated the plasma composition of FAs quantitatively and identified FAs panels, which can distinguish BD from HC (AUC = 0.961), BD‐2w from HC (AUC = 0.978), and BD‐2w from BD (AUC = 0.891) as well. To our knowledge, this is the first study to report the effect of rTMS combined with quetiapine and mood stabilizer intervention on plasma FAs in patients with BD. These results may help the clinical diagnosis of bipolar depression and explain the peripheral molecular mechanism for the acute intervention of rTMS combined with quetiapine and mood stabilizers.

BD is likely caused by the complex interaction of multiple factors, including genetics, neurodevelopment and environmental factors, psychological function, brain networks, hypothalamic–pituitary–adrenal (HPA) axis and gut–brain axis (Harrison, Geddes, and Tunbridge 2019; Li et al. 2022; Patel et al. 2021; Shintani et al. 2023). Family history is the key risk factor for BD. The risk of BD in first‐degree relatives is about eightfold higher than that in the baseline population (Song et al. 2015), and adoption and twin studies also indicated the heritability of BD estimates of approximately 60%–80% (Althoff et al. 2005; Lichtenstein et al. 2009). However, the genetic risk for BD seems to be spread across numerous common variants (Mullins et al. 2021), whereas polygenic risk lacks populational representation and it also interprets with gene–environment correlations (Mostafavi et al. 2020), which limits the application of genetic markers in diagnosing BD. On the other hand, the neuroimaging analysis found broad but minor reductions in the thickness of the temporal, parietal, and frontal cortex (Hibar et al. 2018). Diffuse tension imaging also found the integrity of white matter in the entire brain is generally reduced, especially in the bilateral cinguli and corpus callosum (Favre et al. 2019). Although these findings are highly replicable, they have not yet been clinically applied, mainly because they reflect differences at the group level and the cross with other psychiatric disorders (Nunes et al. 2020; Opel et al. 2020). Considering disturbance in peripheral blood inflammation, oxidative stress, metabolites, and neurotrophins was related to disease activity and neuroprogression in BD (Munkholm, Vinberg, and Kessing 2016; Quintero et al. 2019; Rodrigues et al. 2022), it may be of positive significance to observe the characteristic molecular changes in the blood of patients with BD and establish diagnostic biomarkers.

rTMS mainly demonstrated a positive effect on the treatment of depression (Sackeim 2016), whereas the efficacy of rTMS on BD was inconsistent. A meta‐analysis indicates the response rates of rTMS on bipolar depression were higher than that of sham (Nguyen et al. 2021). However, the blinded, randomized, sham‐controlled literature using rTMS in BD is limited (Konstantinou et al. 2022; Nguyen et al. 2021), and a randomized clinical trial found no evidence for the antidepressant efficacy of iTBS when compared with sham by using clinician‐rated depressive symptoms (McGirr et al. 2021). The discrepancy might be mainly related to the heterogeneity among the study designs, patients and control groups recruited, the course of illness, previous treatments, and small study sample sizes. Considering that the main purpose of this study is to observe the changes in blood FAs metabolism before and after effective treatment. We combined rTMS with pharmacotherapy and found that the combined intervention for 2 weeks effectively alleviated the symptoms related to BD (manifested by a significant decrease in HAMA, HAMD, and BRMS, Table 3), suggesting that the combination of rTMS and pharmacotherapy may have a certain therapeutic effect on bipolar depression. However, due to the lack of a separate drug intervention group, we do not know whether the effectiveness of this combination therapy is better than monotherapy.

FAs are important sources of energy, they also influence neuroinflammation, oxidative stress, and neurotrophins (Morant‐Ferrando et al. 2023; Zhuang et al. 2022; Ziaei et al. 2024). Among them, SCFAs are generally produced by the fecal microbiota and play a role in the function of gut–brain axis. SCFAs can regulate the synthesis of neurotransmitters, tryptophan metabolism, and activity of the HPA axis (Erny et al. 2015; Yano et al. 2015). Previous studies found that SCFAs affect recognition and emotional states (Aho et al. 2021), and levels of SCFAs were correlated with the severity of depressive symptoms (Burton et al. 2023; Skonieczna‐Zydecka et al. 2018). However, the overall alternations of SCFAs in patients with BD are unclear. In the present study, we found that most SCFAs levels, including acetic acid, isovaleric acid, propionic acid, isobutyric acid, and valeric acid, as well as levels of total SCFAs, were decreased in both BD and BD‐2w groups, indicating the decrease of the above SCFAs might be involved in the pathogenesis of BD, but it cannot reflect the effectiveness of rTMS combined with pharmacotherapy. Importantly, we found that levels of butyric acid were normalized and levels of caproic acid were increased after 2 weeks of intervention. Meanwhile, levels of caproic acid were also negatively correlated with scores of HAMA. Butyric acid has anti‐inflammatory and antioxidant properties (Zhou et al. 2023), and it could elevate levels of brain‐derived neurotrophic factor (BDNF) in specific brain regions and relieve depressive‐like behaviors in animal models of depression (Schroeder et al. 2007; Wei et al. 2015). Caproic acid also has immunomodulatory properties, a recent study found that the increase of butyric acid and caproic acid was negatively related to intestinal permeability and inflammation (Liu et al. 2022). Together, these results suggest that the plasma butyric acid and caproic acid might alleviate BD symptoms through their immunomodulatory properties, and the increased butyric acid and caproic acid might be potential indicators for the efficacy of rTMS combined with pharmacotherapy.

MLCFAs were classified as SFAs, MUFAs, and PUFAs and played a role in regulating inflammation, brain structure, and function. Previous studies found that intake of SFA induced depressive‐like behaviors while intake of MUFA promoted signal transduction of neurotransmitters in animals (Sartorius et al. 2012; Sharma and Fulton 2013). Clinical studies already investigated the involvement of MLCFAs in BD. Pomponi et al. found that plasma DHA was decreased while ALA, AA, and C20:5n3 (EPA) were increased in bipolar patients (Pomponi et al. 2013). Evans et al. found that PUFAs intake, and plasma concentrations of PUFAs, including C20:2n6 (eicosadienoic acid, EDA), C18:2n6 (linoleic acid, LA), and EPA, were decreased in bipolar individuals when compared to HCs (Evans et al. 2014). Moreover, patients with BD exhibited abnormal elevations in 18:0 (stearic acid), C16:0 (palmitic acid), LA, AA, and DHA, and reductions in 18:1n9 (oleic acid) in postmortem superior temporal gyrus (McNamara et al. 2014). Here, we found that levels of C22:5n6 and C22:4 were increased whereas levels of C10:0, C17:0, C8:0, LA, total PUFAs, and total N6 PUFAs were decreased in BD than those in the HC group. However, no significant differences between the BD and HC in MUFAs were observed, suggesting that there is an insufficient correlation between plasma MUFAs composition and BD. These discrepancies may arise from the differences in dietary habits, age, gender composition, and sample size among the included subjects. Surprisingly, a large number of MLCFAs, such as C20:5n3 and C22:5n6 as well as total SFAs and MUFAs, were increased in the BD‐2w group when compared with the HC or BD group, indicating that the upregulated peripheral MLCFAs might be potential prediction indicators for rTMS combined with pharmacotherapy.

Considering the diagnosis and treatment of BD still mainly rely on subjective clinical exercises rather than any biological markers nowadays (Nierenberg et al. 2023), we also developed FA panels that could distinguish BD from HC, BD‐2w from HC, and BD from BD‐2w. Notably, SCFAs mainly consisted of the panel that could distinguish BD and HC, while SFAs account for half of the panel that can distinguish BD and BD‐2w, the potential mechanism of the influence of rTMS or pharmacotherapy on plasma FAs is still unreported and needs to be further investigated. Importantly, in the molecular marker panel that distinguishes the BD and HC groups, the levels of eight molecules are related to the level of LDL. Among the molecular marker combinations that distinguish BD and BD‐2w groups, the levels of seven molecules were positively correlated with levels of TG and CHOL. Given that both FAs and antipsychotics combined with mood stabilizers can regulate lipid metabolism (Fushimi et al. 2006; Kohler‐Forsberg et al. 2023), it is difficult to determine the causal relationship between changes in FAs and blood lipid levels.

Nevertheless, several limitations of the present study need to be clarified. First, the enrolled number of patients was limited. We did not distinguish patients with bipolar type I and type II, and we also did not set the validation queue, the alternations of plasma FAs content for different kinds of BD and its relationship with diagnosis and treatment efficacy still need to be further validated through large‐scale studies. Second, we did not strictly limit the daily dosage of quetiapine used, and patients received different kinds of mood stabilizers, including magnesium valproate, lithium carbonate, or sodium valproate, the impact of various drug combinations on FAs is still unclear. Similarly, considering the high rate of treatment failure of monotherapy (Bohlken et al. 2021), we did not set up a monotherapy treatment group, it is unclear whether there is a synergistic effect among rTMS, quetiapine, and mood stabilizers. Moreover, we did not control the patient's diet during hospitalization and the patient is not a first‐episode, the influence of diet and previously used drugs or other treatment methods (such as psychotherapy) on plasma FAs cannot be excluded. Finally, the causal relationship between changes in plasma FAs and symptoms of BD is still unclear, and the therapeutic effect of combining exogenous MLCFAs with traditional treatment or improving gut microbiota by probiotics to regulate SCFAs on BD needs to be further investigated.

## 5 Conclusion

In summary, we characterized the plasma FAs in adult hospitalized patients with bipolar depression before and after rTMS combined with pharmacotherapy intervention. Moreover, we developed combined FAs consisted panels that could effectively distinguish BD from HC, BD‐2w from HC, and BD from BD‐2w. Our results suggest that rTMS combined with pharmacotherapy may play a role in the treatment of BD by regulating peripheral FAs metabolism, which may provide theoretical data for future improvement of BD treatment strategies. Meanwhile, our results also provide a basis for developing diagnostic methods and reveal the potential relationship between plasma FAs and bipolar depression.

## Author Contributions

**Huan Yu**: Investigation, writing–original draft, validation. **Xue‐jun Liang**: Investigation, data curation. **Yu‐ting Qiao**: Investigation, validation, data curation. **Lin Guo**: Data curation, investigation. **Zhao‐yang Li**: Data curation. **Cui‐hong Zhou**: Formal analysis. **Wen‐jun Wu**: Investigation, funding acquisition. **Rui Li**: Conceptualization, writing–original draft, writing–review and editing. **Zheng‐wu Peng**: Conceptualization, writing–original draft, writing–review and editing, funding acquisition, supervision.

## Conflicts of Interest

The authors declare no conflicts of interest.

### Peer Review

The peer review history for this article is available at https://publons.com/publon/10.1002/brb3.70341

## Ethics Statement

The research protocol was approved and registered by the Chinese Clinical Trial Ethics Committee (number: ChiECRCT20200090) and Clinical Trial Registry (number: ChiCTR2000032118).

## Supporting information




**SUPPORTING INFORMATION TABLE 1** Clinical data for individuals.

## Data Availability

The data will be made available upon request.
